# A Virtual Reality Intervention to Promote Uptake of Medications for Opioid Use Disorder in the Emergency Department Following Opioid-Involved Overdose Based on Input From People With Lived Experience: Development Study

**DOI:** 10.2196/84397

**Published:** 2026-07-06

**Authors:** Zina Trost, Andrea Stevenson Won, Hari Prasad Naidu Boyapati, Keri Costa, Josh Davenport, Gail D’Onofrio, Leslie Drager, Phillip Corey Shum, Michael Wolfe Pierce, Liana Fraenkel, William Soares III

**Affiliations:** 1 Department of Psychological and Brain Sciences, Texas A&M University, College Station, TX, United States; 2 Department of Communication, Cornell University, 237 Mann Drive, Suite 492, Ithaca, NY, 14850, United States, 1 9199060287; 3 Berkshire Medical Center, Pittsfield, MA, United States; 4 Immersive Experience Laboratories LLC, Birmingham, AL, United States; 5 Yale School of Medicine, New Haven, CT, United States; 6 Yale School of Public Health, New Haven, CT, United States; 7 Department of Emergency Medicine, Baystate Medical Center, Springfield, MA, United States

**Keywords:** virtual reality, VR, opioid use disorder, opioid agonist medication treatments, patient and public involvement, development

## Abstract

**Background:**

Opioid use disorder (OUD), which leads to thousands of deaths per year, continues to pose a tremendous challenge to health care systems. While medications for opioid use disorder (MOUDs), such as buprenorphine and methadone, are the most effective treatments for OUD, the initial patient conversation and decision to engage in MOUDs remain major barriers. Decision-making is strongly influenced by the patients’ current emotional state. While the emergency department (ED) is a promising access point for initiating MOUDs, patients’ readiness to engage in treatment can be limited, in part, by the negative emotions and experiences associated with the ED visit.

**Objective:**

We co-designed and developed a novel emotion-based virtual reality (VR) intervention to increase patient readiness to engage in shared decision-making for treatment with MOUDs following an ED admission for an opioid-related overdose.

**Methods:**

This paper describes a 3-stage, iterative process involving 59 stakeholders, including people with lived experience (LE), to design and develop an intervention that resulted in a VR app and preliminary assessments of user experience.

**Results:**

Our process incorporated the perspectives of LE stakeholders across all aspects of the design and refinement of the VR platform VR-Choice. VR-Choice consists of a VR journey up a mountain (“Mountain Journey”), culminating in the presentation of nine 180-degree videos showing different positive aspects of life during recovery (“Possible Worlds”). Of the 20 participants who experienced the final platform version, none reported more than slight motion sickness symptoms. Nineteen of the 20 participants stated they liked the videos shown in the final version, considered it to be potentially helpful for patients in the emergency room after an opioid-involved overdose, and thought the experience should be offered to those patients. All participants agreed that the program was easy to use, and only 1 participant described it as confusing.

**Conclusions:**

To our knowledge, this is the first effort to use feedback from participants with LE to design an engaging VR experience for people in the ED who may be candidates for MOUDs. Participants’ feedback was positive, and we will move forward with development.

## Introduction

This paper describes the development of an immersive virtual reality (VR) experience, “VR-Choice,” designed to increase patient readiness to engage in shared decision-making (SDM) for medications for opioid use disorder (MOUDs) treatment in the emergency department (ED). The VR intervention was developed for use by trained hospital staff in collaboration with ED clinicians.

While deaths related to drug overdoses have recently decreased, opioid use disorder (OUD) remains a leading cause of death in the United States, with 79,384 drug overdose deaths occurring in 2024 [1]. The continuing national opioid crisis necessitates novel initiatives aimed at increasing the adoption of effective treatment. In particular, methadone and buprenorphine (MOUDs) are Food and Drug Administration–approved for OUD and are associated with an approximately 50% reduction in mortality [2-4]. MOUDs also decrease the risk of overdose and acute care use compared with nonpharmacologic treatment [2-5]. Additional benefits include increased retention in treatment, reduced criminal behavior, improved employment, better maternal or infant outcomes, and reduced transmission of HIV and hepatitis C [4,6]. There is strong support that the ED represents a critical access point to engage individuals in care and OUD medication treatment [7]. A landmark randomized controlled trial found that 78% of patients randomized to ED-initiated buprenorphine were engaged in treatment at 30 days, compared to 37% referred to primary care and 45% given a brief motivational interview [7]. Bogan et al [8] extended these findings, showing feasibility across 3 ED sites, with 77% attending follow-up and 60% remaining in treatment at 30 days. Despite their effectiveness, the uptake of MOUDs remains extremely low, with only 19.4% of individuals with OUD receiving MOUDs [9]. ED physicians cite low interest in MOUDs as a top barrier to buprenorphine uptake [10,11]. Data from the US National Survey on Drug Use and Health found that among adults with a substance use disorder who did not receive substance use treatment, 95.6% did not *perceive* that they needed treatment [12]. While education regarding the benefits associated with MOUDs may increase perceived need, existing strategies (eg, motivational interviewing, peer coaches) have not been sufficient [13,14]. Decision-making is strongly influenced by patients’ current emotional state. While the ED is a promising access point to initiate MOUDs, patients’ readiness to engage in treatment can be limited, in part, due to the negative emotions and experiences associated with the ED visit.

**Figure 1.**
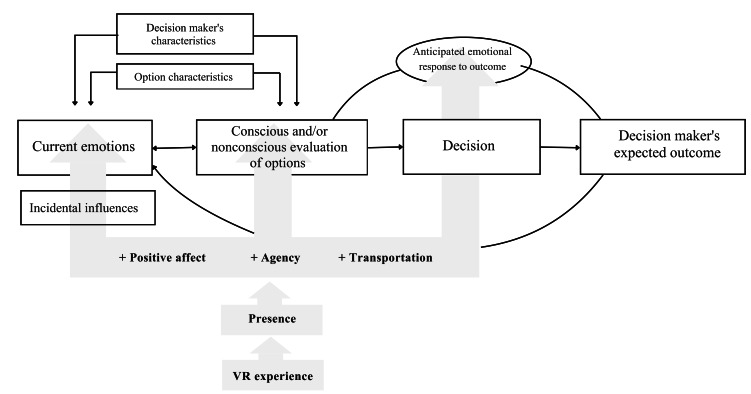
The emotion-imbued choice model [15] with potential mechanisms of VR intervention. VR: virtual reality.

VR-Choice design was informed by the emotion-imbued choice (EIC) model of Lerner et al [15] (Figure 1), which describes the centrality of emotions and affects decision-making. The model posits that an individual’s current emotions affect how options are appraised—for example, a positive mood state increases attention to potential benefits. In contrast, negative cognitive or mood states (anxiety, anger) that characterize many individuals with OUD [16-18] are likely to hinder effective decision-making [19-21]. For instance, fear and anxiety may increase (1) preference for the existing state, even when harmful, due to an exaggerated aversion to change (status quo bias); (2) hasty rejection of treatment options (ie, MOUDs) due to a failure to consider all relevant information (premature closure bias); and (3) the tendency to focus solely on information that confirms pre-existing beliefs (confirmation bias) [19]. Each of these biases hinders patients’ ability to become fully informed. Conversely, positive emotions encourage attention to benefits and may increase the perceived value of MOUDs; thus, an improvement in a patient’s mood may result in greater valuation of MOUDs’ benefits. The model also highlights future-oriented processes: anticipated emotions (eg, imagining feeling better on MOUDs) can enhance evaluations of treatment options. Finally, current emotions and expected outcomes interact bidirectionally, reinforcing each other over time [15,22-25].

Drawing on the EIC framework, VR was conceptualized as a promising modality to enhance receptivity toward SDM for MOUD treatment [26]. Specifically, VR has emerged as an effective technology capable of eliciting intense positive affective states in both healthy and clinical populations [27,28], including among acutely distressed individuals and those experiencing acute physical pain [29-31]. VR has also been used to improve affect and reduce pain and anxiety specifically in the ED setting [32,33]. Relatedly, VR can transport people away from their physical environment by replacing sensory information from the physical world with virtual sensory information [34]. Transportation is particularly important because the characteristics of the external physical (ie, the ED) as well as internal (emotional, physical) environment may be acutely distressing to individuals with OUD. Facilitating virtual transport away from these negative influences is expected to improve affect and enable a more open mindset from which positive decisions can be made [35]. Similarly, drawing on interactivity, VR can support feelings of agency, perceived control, and forward momentum, which are consistently lower in individuals with substance use disorder [36-39]. In this sense, VR-facilitated agency can provide individuals in the ED (ostensibly an environment in which they have little control) with evidence of perceivable sensory effects associated with their own actions and choices. Finally, as a simulative technology, VR can support imagination by enabling the simulation of future experiences (“Possible Worlds,” see below). In this way, VR can help counteract the tendency of patients with OUD to focus on immediate, short-term rewards and to discount future outcomes [40,41].

Accordingly, VR-Choice was developed to leverage the ED as a low-threshold, effective approach to engage individuals with OUD. We describe the development of a curated VR experience to increase patient readiness to engage in SDM, a patient-centered, collaborative process in which providers and patients jointly evaluate options, considering individual needs and preferences to determine a course of treatment.

## Methods

### Overview

VR-Choice operates using the HTC Vive XR Elite headset and Vive Ultimate Trackers for the left and right wrists. The VR-Choice experience has 2 components. First, participants are presented with a calming virtual environment, developed using the Unity 3D gaming platform (version 2022.3.57f1) [42] intended to induce positive affect and an open mindset; we focused on facilitating the emotion of awe, as it involves cognitive processes—appraisals of vastness, feelings of smallness and connectedness, and accommodation of new ideas—that overlap with the mechanisms within empirically supported interventions for substance use disorder (eg, mindfulness [43]). In this environment, participants proceeded through 4 scenes of increasing elevation to the top of a mountain (“Mountain Journey”). Subsequently, participants were presented with an array of virtual bubbles, each of which, when touched, allowed an immersive glimpse of a 180-degree video showing a positive representation of life after recovery (“Possible Worlds”).

**Figure 2.**
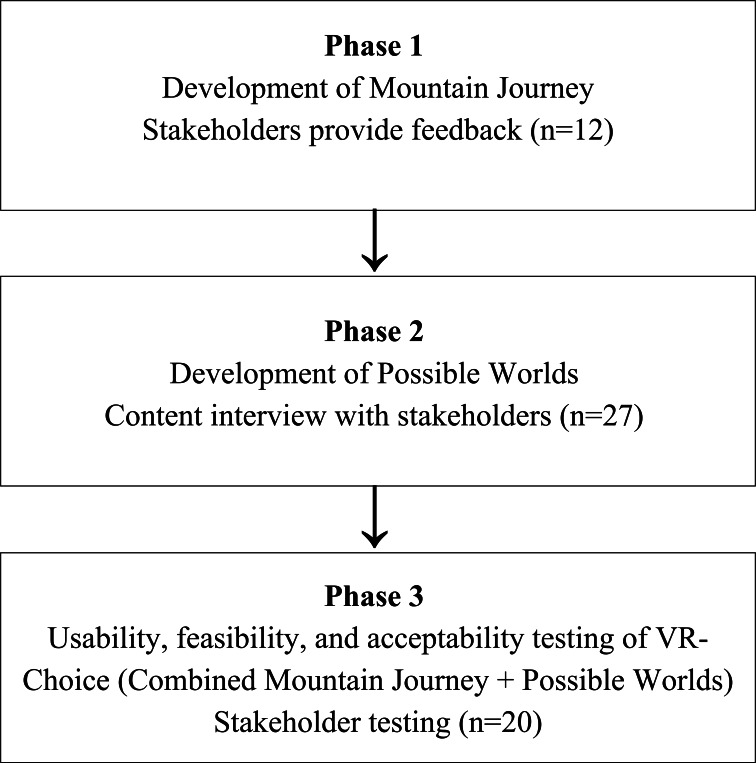
Development of VR-Choice modules for use in the emergency department (ED). VR: virtual reality.

Our process for developing the 2 components of the virtual experience was iterative. In phase 1, we developed the virtual environment for Mountain Journey, which was characterized by relaxation, awe, and forward momentum, modifying it iteratively based on feedback from the research team, academic labs, and interviews with participants with lived experience (LE), who were community members with past or current nonprescribed opioid use. In phase 2, we ideated, selected, storyboarded, and filmed nine 180-degree videos representing positive aspects of life during recovery for Possible Worlds, beginning with interviews with LE stakeholders. In phase 3, we collected feedback on the combined VR-Choice experience (Mountain Journey + Possible Worlds) from LE stakeholders (see *Phase 3 Feedback Process* for a detailed description of measures). Verbal consent was obtained from all potential stakeholders prior to obtaining feedback or audio recording VR sessions. We describe the process for each phase in Figure 2.

### Research Team

The research team consisted of clinicians, researchers, and developers from 4 different institutions, all based in the continental United States. Two members of the team were clinical investigators with expertise in substance use and MOUDs. They received funding for the project and directed the overall research team. Two team members headed VR laboratories specializing in clinically oriented VR interventions. They both engaged their laboratory trainees in the iterative evaluation of the VR environments. The development team consisted of 2 developers with experience in creating VR environments for clinical applications, focusing on pain and rehabilitation. The team met via Zoom weekly or semiweekly during the course of the project and shared stimuli by email and through Google Drive.

### Ethical Considerations

All procedures were reviewed and approved as exempt by the Baystate Medical Center Institutional Review Board, and all participants signed informed consent forms or consented verbally. Participants were compensated US $50 per hour, prorated by time (ie, if the interview lasted only half an hour, the participant received US $25). All data presented have been anonymized.

## Results

### Phase 1: Developing Mountain Journey

#### Phase 1 Participants

Participants in this stage of development included 6 LE stakeholders who took part in Zoom meetings, 6 LE stakeholders who viewed and commented on videos in person, and members of the participating laboratories and development team (Table 1).

**Table 1.** Demographic information of stakeholders with lived experience who provided feedback on Mountain Journey on Zoom or in-person.

Stakeholders	Zoom, n (N=6)	In-person, n (N = 6)
Gender		
Male	2	4
Female	4	2
Self-identified race		
Black	1	4
Hispanic	0	0
White	5	2
American Indian or Alaska Native	0	0
Time in recovery from OUD^a^		
Currently using drugs	0	0
No drug use for 1-3 months	0	3
No drug use for 4-12 months	0	1
No drug use for 1-2 years	0	1
No drug use for 2-5 years	1	1
No drug use for over 5 years	2	0
Unknown	3	0

a OUD: opioid use disorder.

#### Phase 1 Feedback Process

##### Overview

Once the initial virtual imagery was programmed, participant feedback was sought in 2 ways. First, the research teams met with core LE stakeholders (n=6) over a series of 4 Zoom meetings. During these meetings, a video of the virtual scenery was shared and discussed with all the participants, and recommendations were made for further iteration in terms of journey stages, content, and pacing. LE stakeholders did not have VR equipment at this early point in the development process; however, equipment was available to members of the laboratories of authors ASW and ZT to explore navigation options. Following agreement on content and navigation, the Trost laboratory team collected in-person feedback from additional on-site LE stakeholders (n=6, data shown in Table 1), and feedback on content and navigation was iteratively incorporated as described below. It was not feasible to engage the same stakeholders repeatedly across all phases of the study.

##### Phase 1 Navigation

**Figure 3.**
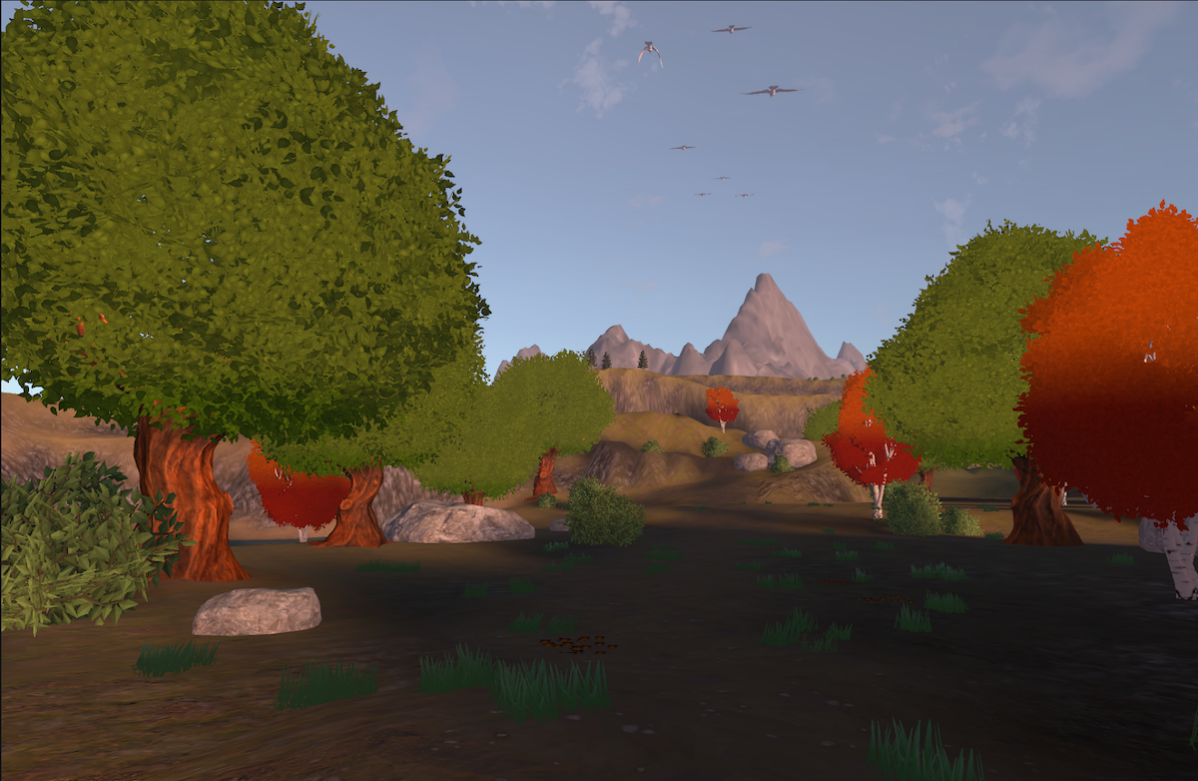
This shows the opening scene of the environment. Birds fly overhead, directing users toward the mountain.

Given the environmental constraints (eg, attached medical equipment) and the sensitive physical condition of the target population, considerable discussion focused on the pacing of these scenes and the method by which participants would progress up the mountain. Several methods were tested, including joystick navigation and transportation or teleportation to successive points along the ascent. The various ascent and pacing options were tested successively by students in the research labs, as well as by an LE stakeholder. To alleviate concerns about motion sickness and prevent participant confusion, we settled on a timed scene-fade approach that ensured all participants advanced through the mountain stages at the same pace. The opening scene faded to a scene one-third of the way up the mountain, a scene two-thirds of the way up the mountain, and the final scene at the mountain top (Figures3^6^). As participants progressed up the mountain, snow fell more heavily (to indicate increasing elevation), and the music became louder.

**Figure 4.**
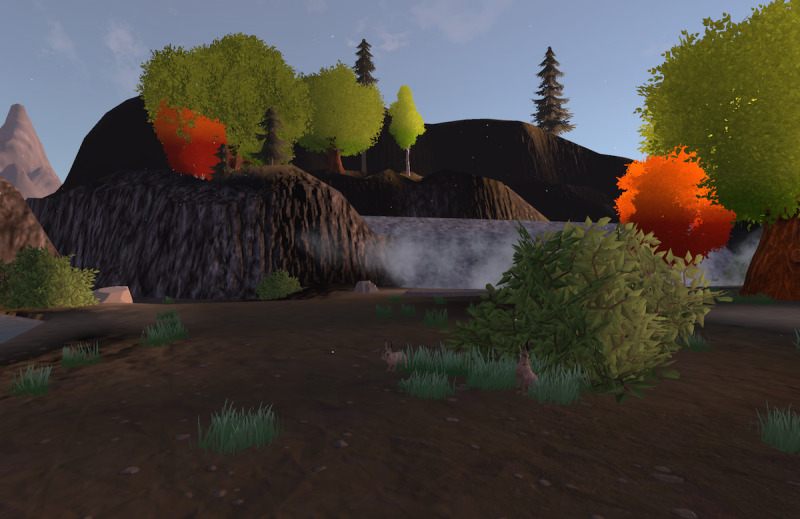
The second scene introduces rabbits browsing in the foreground of the scene.

**Figure 5.**
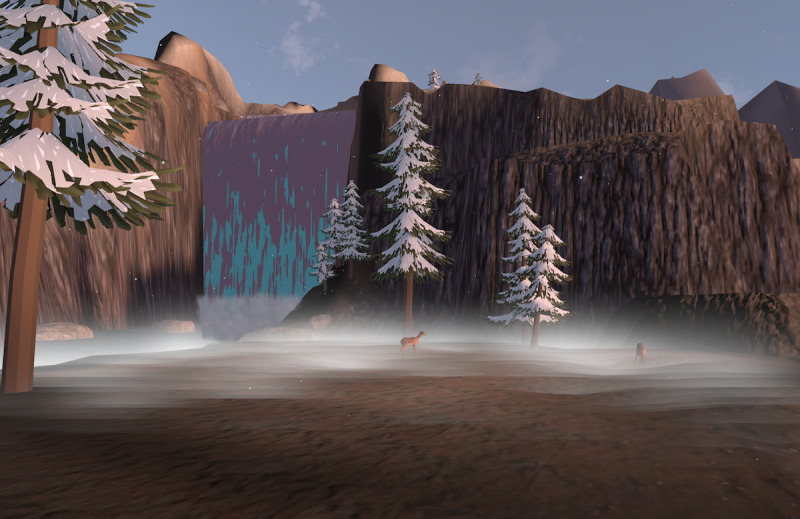
This shows the third scene, in which the snow is more prominent, indicating a higher altitude. Deer are browsing near the waterfall.

**Figure 6.**
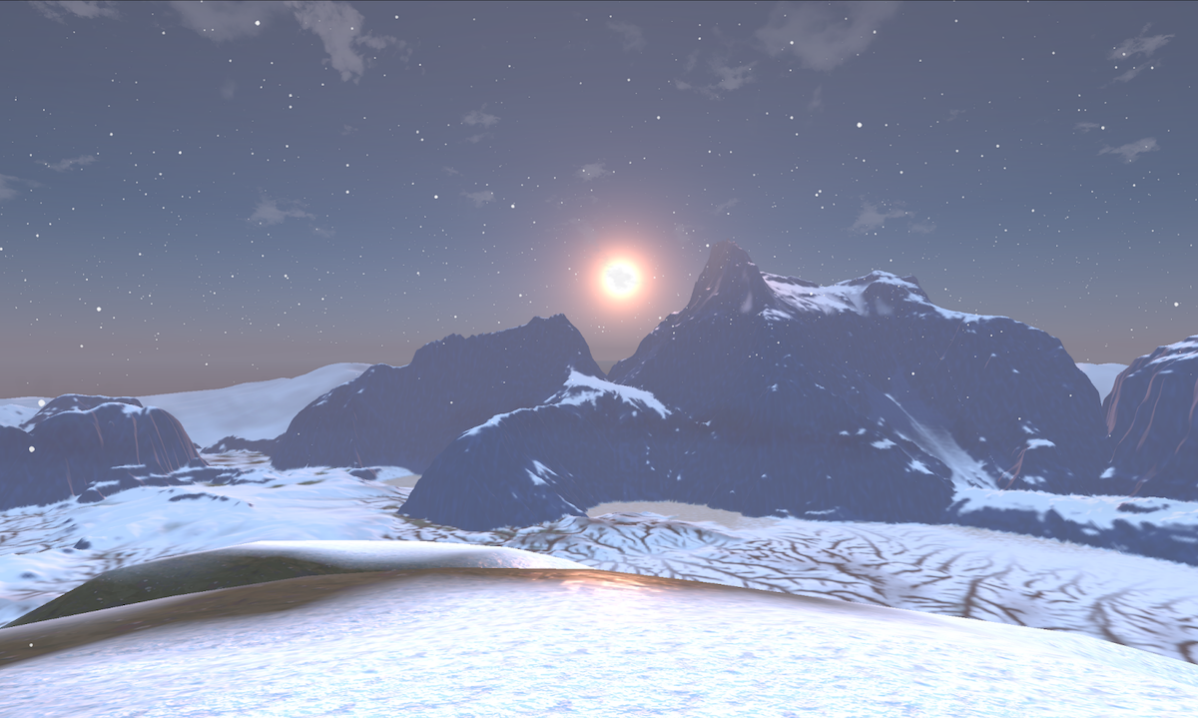
This shows the scene at the top of the mountain.

##### Phase 1 Content

The initial content for Mountain Journey was collaboratively developed by members of the research team. To generate content for Mountain Journey, the research team searched the literature to identify previous representations of awe in VR, which were limited. We were then provided with existing 360-degree VR imagery, which had been validated to elicit feelings of awe in nonclinical populations [44,45]. Second, we searched for and identified awe-inspiring music, which was likewise specifically validated in the literature [46] to be used as the background for the intervention. Due to concerns about space on the headset, the designers selected a more abstract, lower-resolution style for the mountain scenes. The app was developed using Unity 2019 and later upgraded to Unity 2022 for performance and analytics enhancements. Gaia (Unity asset) was used for terrain generation within the engine to speed up the process of assigning materials to denote terrain height and slope, as well as to generate a procedural sky for “time of day” manipulation. GeNa (Unity asset) was used to lay out rivers and paths with splines. Vulkan rendering was implemented in place of OpenGL to improve performance and reduce draw calls through object instancing. Other optimizations included reducing texture sizes and video bitrate to achieve a balance between performance, image integrity, and file size. Ideally, we wanted to achieve a framerate that would reduce the risk of nausea for the user while also presenting an open and expansive outdoor environment. In discussions, LE stakeholders emphasized the need for a period of initial relaxation and orientation to the VR space in order to counteract the effects of a busy ER or existing anxiety. Early discussion also emphasized avoiding direct exhortations toward recovery during the VR experience, instead focusing on participants’ emotional experience. LE stakeholders also requested the addition of movement and animals in the virtual scenes to keep the participants’ attention. These elements were iteratively added. Methods of providing instructions (visible and spoken words) were discussed. The final script can be found in Multimedia Appendix 1. Mountain Journey development resulted in a 2-minute, 35-second-long experience, with approximately 25 seconds at each scene following the initial orientation, as participants proceeded up the mountain.

### Phase 2: Developing Possible Worlds

#### Phase 2 Participants

Participants in this part of development consisted of LE stakeholders who were interviewed to generate bubble content and participated in Zoom meetings to provide feedback on the storyboards, members of participating laboratories, and the development team.

To optimize geographical diversity, the Baystate and ZT laboratories first conducted interviews with LE stakeholders (n=27) in-person, via phone, and on Zoom (relative to stakeholder availability) in order to generate relevant content for the 180-degree videos that would appear in the bubble scenes described above. One member of the research team was bilingual and served as a translator for 3 individuals who were interviewed in Spanish. In total, stakeholders included 10 women and 17 men, ranging in age from 21 to 71 years old (mean 50.30, SD=11.84). Thirteen stakeholders identified as Black or African American, 8 identified as Hispanic, and 6 as White. Recovery ranged from less than 1 month (n=4) to over 11 years (n=5). Demographic information, including recovery characteristics, is outlined in Table 2.

**Table 2.** Demographic information of stakeholders with lived experience who provided suggestions for the 180-degree video content for Possible Worlds.

Stakeholders	Participants, n
Gender	
Male	17
Female	10
Self-identified race	
Black	13
Hispanic	8
White	6
American Indian or Alaska Native	0
Time in recovery from OUD^a^	
Currently using drugs	4
No drug use for 1-3 months	4
No drug use for 4-12 months	4
No drug use for 1-2 years	1
No drug use for 2-5 years	6
No drug use for over 5 years	7
Unknown	1

a OUD: opioid use disorder.

#### Phase 2 Feedback Process

Stakeholders were introduced to the concept and purpose of Possible Worlds and were invited to suggest future situations that might be hopeful or motivating for recovery (eg, “What would you look forward to when you are in recovery from your substance use disorder and not using drugs?”). A minority of participants suggested depicting the negative consequences of opioid use, with the idea that these would motivate people toward recovery. Such negative suggestions included depictions of jail and overdose. Because our aim was to calm and empower participants, we decided to depict only positive situations.

Following these interviews, the collected feedback was reviewed over Zoom with the research team and LE stakeholders. Three principal themes emerged: belongingness, physical and financial security, and a physical transformation to a healthy body. The subsequent Zoom discussions with the videographer focused on how to best represent the selected themes visually in the VR environment. Storyboards were iteratively presented and discussed, and each recorded scene was reviewed by the entire research team.

#### Content: Possible Worlds Scenes

**Figure 7.**
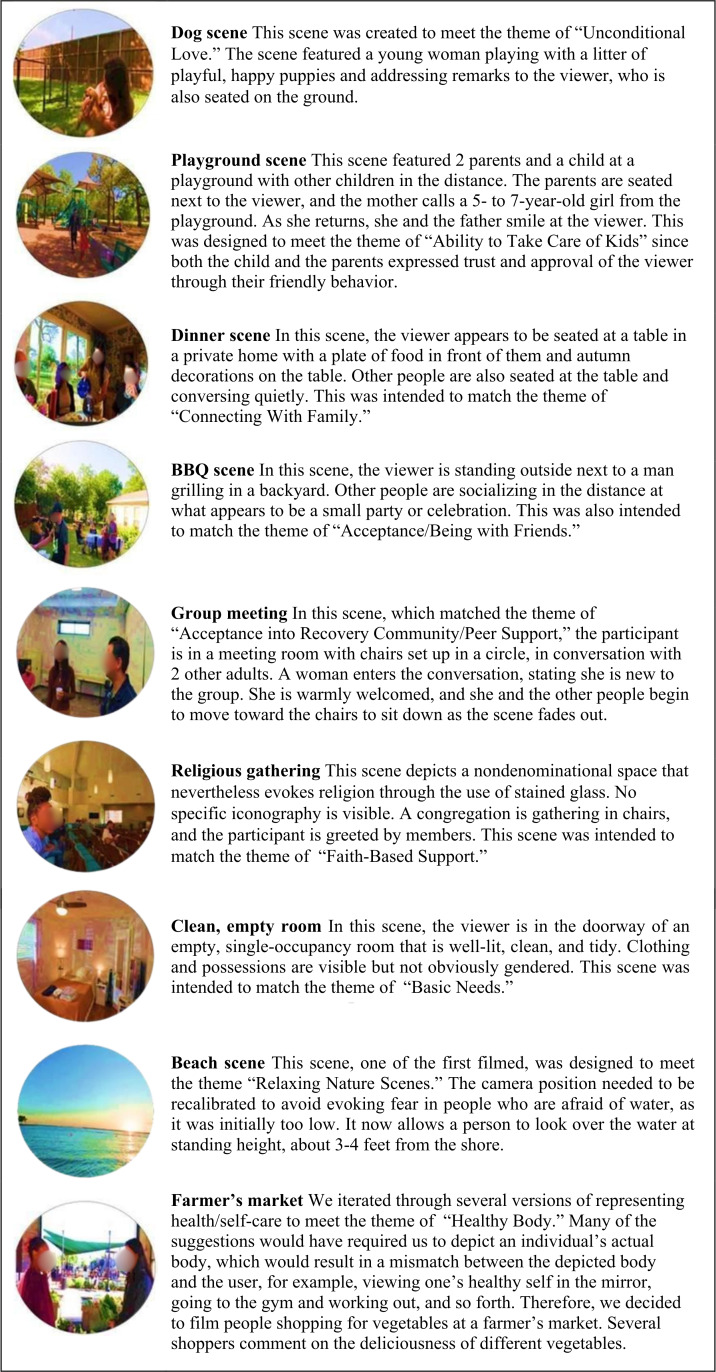
A screenshot from each video offered to participants is shown next to a description of the video itself. Barbecue:

All videos were shot in 4K and were 180 degrees. In other words, participants could turn their heads 90 degrees to the left or the right. If they tried to turn completely around (unlikely in the VR environment), they would see only black space. Although the user’s body was not represented in the VR (when a user looked down, they would only see blank space), the camera was positioned in each scene to create the illusion of embodied presence in the environment. For example, the camera was positioned at an average seated or standing height, in a position where a person could plausibly be located (eg, in a chair, rather than embedded in an object or the ground). Actors in scenes were directed to maintain plausible social distance and make “eye contact” with the camera, so that viewers would feel they were being personally addressed. Although users’ individual bodies were not represented, we still needed to consider how to represent diversity through scenes and actors, bearing in mind that individuals’ experiences would vary. Because there was no avatar or other way to provide users with a visible body, we selected scenes where the body of the participant could be “invisible” without evoking uncanniness. Videos ranged from 32 to 40 seconds each (Figure 7).

### Navigation

In addition to the content and composition of the videos, we also discussed how participants would navigate this part of the experience. The bubbles were laid out in a grid pattern on a slightly concave curvature to allow for easier viewing and reachability. The bubble videos are rendered at 720p and compressed to 360p on a slightly transparent material to allow for a less claustrophobic presentation while keeping the file sizes and processing overhead low enough to run 9 videos simultaneously without catastrophically plunging the framerate. Vignettes of each scene were visible within each “bubble” so that participants could preview the material and avoid selecting content that might be distressing for personal reasons. For example, some participants might choose to avoid viewing materials with children, animals, or religious imagery. Participants could select bubbles more than once and could view as many of the videos as they liked. A voiceover at the beginning of the scene suggests that participants view at least 3 bubbles and that they could leave any scene at any time.

### Phase 3: Testing the Usability, Feasibility, and Acceptability of the Combined VR-Choice App

#### Phase 3 Participants

Participants for this part of the development were LE stakeholders who were recruited from a local transitional housing site serving people with OUD and from a nonprofit community health prevention and education drop-in center that provides health screenings as well as opioid-involved overdose prevention training, education, and naloxone distribution. All LE participants in this portion of the study had a past or current history of OUD and overdose.

This VR-Choice app was tested with an additional group of 20 LE stakeholders. Stakeholders included 13 men and 7 women, ranging in age from 33 to 72 years old (mean 51.80, SD 11.96). Ten stakeholders identified as Black or African American, 2 identified as Hispanic, 7 as White, and 1 as American Indian or Alaska Native. Recovery status ranged from currently using drugs to over a 5-year duration. Recovery characteristics are outlined in Table 3. Of the participants, 1 endorsed regular VR use, 10 reported having tried VR a few times, and 9 indicated no VR experience.

**Table 3.** Demographic information of 20 stakeholders with lived experience who provided feedback on the combined VR-Choice experience that included both Mountain Journey and Possible Worlds.

Stakeholders	Participants, n
Gender	
Male	13
Female	7
Self-identified race	
Black	10
Hispanic	2
White	7
American Indian or Alaska Native	1
Time in recovery from OUD^a^	
Currently using drugs	2
No drug use for 1-3 months	4
No drug use for 4-12 months	3
No drug use for 1-2 years	3
No drug use for 2-5 years	4
No drug use for over 5 years	4
Unknown	0

a OUD: opioid use disorder.

#### Phase 3 Feedback Process

**Figure 8.**
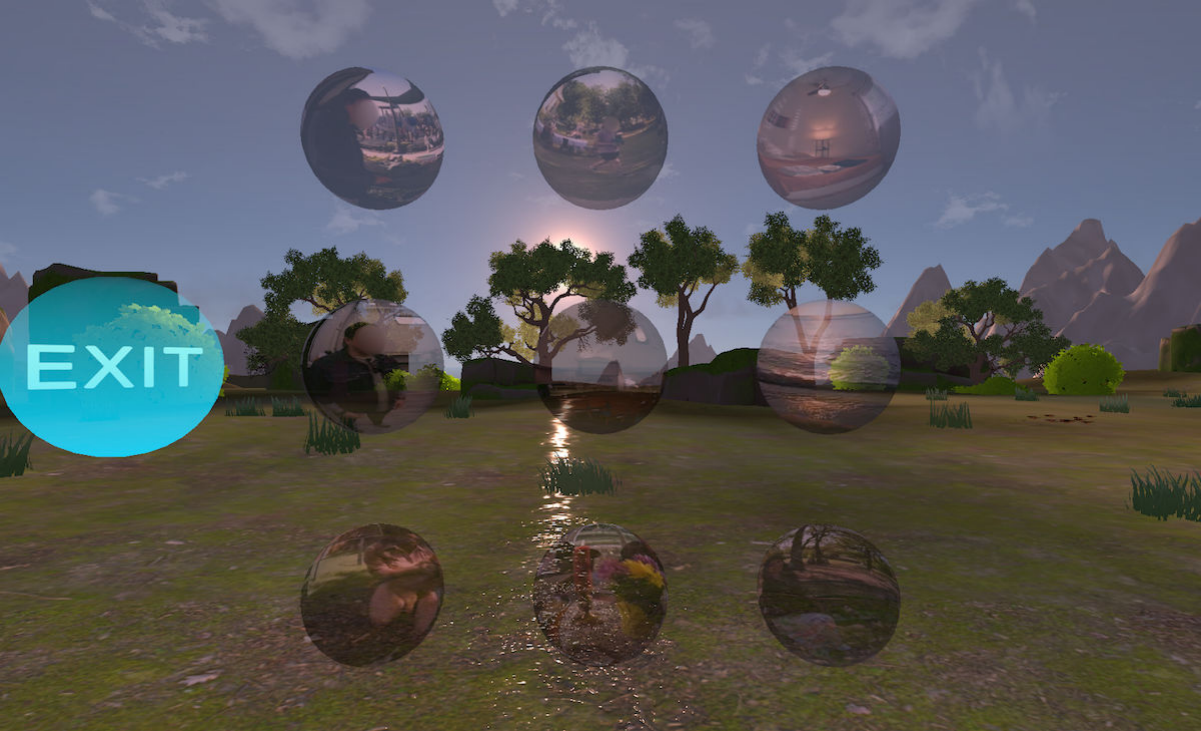
The bubbles presenting the video content appeared in groups of 3, with a new set of videos being added after participants had watched at least 1 from each group. This figure shows the final array of videos.

The final VR-Choice app combined the Mountain Journey and Possible Worlds sections. Following the ascent up the mountain, participants are automatically transported back to the orientation location at the beginning of Mountain Journey, where they are gradually presented with a grid of 9 bubbles (Figure 8). Three bubbles appear in front of the participant, and after they choose and view one, another set of 3 bubbles enters the scene. In both written and spoken instructions, participants were asked to select at least 3 bubbles, with no upper limit on the number they could choose.

After experiencing the VR-Choice app, participants completed several measures assessing simulation sickness, usability, and acceptability. The following measures were employed, and the results are summarized in Table 4. With the exception of usability items, which were specific to the content of VR-Choice, all measures had previously been validated in VR-related intervention research.

1. Simulation sickness: Participants also responded to 9 items drawn from the Simulation Sickness Questionnaire [47], inquiring about VR-related symptoms, such as vertigo, blurred vision, and eye strain. Items were rated on a 4-point scale with response options ranging from not at all (0), slightly (1), moderately (2), to very (3).
2. Usability: Usability included specific questions about navigation (“I chose which items I wanted to experience”, “I controlled my actions and experiences in the bubble space”, and “I chose how many different bubbles I wanted to experience”) and general ease of use (“I found this VR program easy to use” and “I think this VR program was confusing”). Items were rated on a scale of 1 (strongly disagree) to 5 (strongly agree).
3. Acceptability: Acceptability items were drawn and adapted from the Treatment Evaluation Inventory [48] and included “I think this VR program is likely to help patients in the Emergency Room after an overdose”, “I like the scenes in the bubbles in the VR program”, and “I think this VR program should be offered to patients in the Emergency Room after an overdose.” Items were rated on a scale of 1 (strongly disagree) to 5 (strongly agree).

A few participants endorsed items from the Simulation Sickness Questionnaire (Table 4), but all were characterized as being “Slight.” In terms of the usability of the experience of interacting with bubbles, 9 agreed and 11 strongly agreed with “I chose which bubbles I wanted to experience.” Similarly, 10 agreed, and 10 strongly agreed with “I chose how many different bubbles I wanted to experience.” Responses to “I controlled my actions and experiences in the bubble space” were more varied, with 9 agreeing, 8 strongly agreeing, 2 being neutral, and 1 disagreeing.

Five participants agreed, and 15 strongly agreed with the statement “I found this program easy to use”; similarly, 3 disagreed, and 16 strongly disagreed with the statement “I think this program was confusing”—1 person agreed with this statement. In terms of acceptability, 12 individuals strongly agreed with the statement “I like the scenes in the bubbles in the VR program”; 6 agreed, 1 was neutral, and 1 disagreed. Thirteen people strongly agreed with the statement “I think this VR program is likely to help patients in the Emergency Room after an overdose”; 6 agreed, and 1 was neutral. Similarly, 15 people strongly agreed with the statement “I think this VR program should be offered to patients in the Emergency Room after an overdose”; 4 agreed, and 1 was neutral.

**Table 4.** Participant responses to survey measures.

Domain	Participants, n
Simulation sickness^a^	
General discomfort	2
Fatigue	0
Eye strain	3
Difficulty focusing	2
Headache	1
Fullness of the head	3
Blurred vision	3
Dizziness	1
Vertigo	1
Usability	
“I chose which bubbles I wanted to experience”	
Strongly agree	11
Agree	9
“I chose how many different bubbles I wanted to experience”	
Strongly agree	10
Agree	10
“I controlled my actions and experiences in the bubble space”	
Strongly agree	8
Agree	9
Neutral	2
Disagree	1
“I found this program easy to use”	
Strongly agree	15
Agree	5
“I think this program was confusing”	
Agree	1
Disagree	3
Strongly disagree	16
Acceptability	
“I like the scenes in the bubbles in the VR^b^ program”	
Strongly agree	12
Agree	6
Neutral	1
Disagree	1
“I think this VR program is likely to help patients in the Emergency Room after an overdose”	
Strongly agree	13
Agree	6
Neutral	1
“I think this VR program should be offered to patients in the Emergency Room after an overdose”	
Strongly agree	15
Agree	4
Neutral	1

a All symptoms of simulator sickness were labeled as “Slight.”

b VR: virtual reality.

## Discussion

### Principal Findings

This paper describes a 3-stage, iterative process that drew heavily on incorporating the experiences and perspectives of people with LE in all aspects of the design and refinement of the VR-Choice platform. VR-Choice was intended to address affect and motivation among patients admitted to the ED following overdose, with the goal of increasing their readiness to engage in SDM for treatment with MOUDs. VR-Choice consists of a VR journey up a mountain (“Mountain Journey”) culminating in the presentation of nine 180-degree videos showing different positive aspects of life during recovery (“Possible Worlds”). The bubble content reflects themes derived from LE interviews conducted across the country. In total, we were able to engage with 59 participants with past or current OUD during the design process. Of the 20 participants who experienced the final version of the platform, none reported more than slight motion sickness symptoms. Nineteen of 20 participants stated they liked the videos shown in the final version, considered it to be potentially helpful for patients in the emergency room after an opioid-involved overdose, and thought the experience should be offered to those patients. All participants agreed that the program was easy to use, and only 1 participant described it as confusing.

Both the context and patient population targeted by VR-Choice necessitated careful consideration of multiple ethical and design factors. Specific efforts were made to mitigate simulation sickness, given the likely physical state of patients following overdose or overdose treatment, which informed the design of transitions “up the mountain” and between scenes. Once the intervention is deployed clinically, participants will experience the virtual environment seated and under the observation of health care workers. The positive feedback from non–ED-based OUD stakeholders is encouraging but will need to be further examined within the specific context of postoverdose ED care.

In order to mitigate the risk of psychological distress in a vulnerable population, substantial attention was given to the content of Possible Worlds bubbles, balancing the inclusion of themes endorsed as meaningful by stakeholders (eg, caring for children) with sensitivity to content that could be personally triggering. To that end, the content of each bubble is made readily apparent to viewers (visible inside the bubble) to inform participants’ selection, and mechanisms were incorporated to allow for a quick and easy exit if desired. All content was carefully curated to be positive. While the development of the administration protocol draws heavily on input from LE experts, including individuals with OUD and ED personnel, issues around informed consent remain relevant given the potential differences in states of readiness among ED patients during an opioid-related visit. For future evaluations of VR-Choice involving patients in the ED, the informed consent process will include a detailed screening to ensure patients are not altered in mental status, feeling sick, or in significant distress prior to the experience. Consent will also include an overview of VR-Choice—including the content of the Possible Worlds bubbles—and a clear emphasis that patients may remove the headset and stop the experience at any time, for any reason. Following preliminary evidence supporting the feasibility and efficacy of VR-Choice within the ED, future large-scale studies should incorporate implementation metrics, including the integration of VR-Choice within ED workflows and evaluation of the intervention’s cost-effectiveness.

### Limitations

This experience was limited by both technical factors and constraints in the co-design process. In terms of technical limitations, there was no interactivity, personalization, or embodiment in any of the VR or 180-degree video scenes. Participants could not reach out and touch or manipulate objects. In the video scenes, while we did our best to simulate a sense of social presence, the actors were only pretending to make eye contact and engage with an imaginary speaker and as such could not respond to any behavior on the part of the participants. Because the camera was set at a height of 5′5″, it did not necessarily match participants’ actual height. Similarly, neither hands nor body were portrayed in any of the scenes to avoid a mismatch with participants’ actual bodies. Therefore, when participants looked down, they would see only empty space. As technology continues to advance, we expect to eventually gain the ability to rapidly create avatars of a user and integrate them into digital scenes; current platforms capable of this, such as the Apple Vision Pro, remain prohibitively expensive and technically demanding for the purposes of this project.

In addition, there were challenges in working with our target population. First, obtaining a representative sample of participants with LE was especially challenging. Participants were often difficult to contact, so follow-up interviews were challenging to conduct. Second, many people do not have personal experience with VR, meaning that for many stakeholders, including many clinical members of the research team, it was challenging to imagine possible outcomes. Future work developing in this area could consider how to provide a sample VR experience before asking people to comment on screenshots or other nonimmersive content. However, this brings other issues, including the fact that the novelty of participants’ “first VR experience” might create an anchoring effect.

### Future Directions

Next steps will include feasibility, acceptability, and preliminary efficacy testing in the actual ED setting with the population of interest (individuals following opioid-involved overdose). The data collected from this work will subsequently inform the planning of a larger randomized controlled trial testing the VR-Choice intervention against a standard intervention type.

Future studies may further explore mechanisms of change and participant responses, drawing on both EIC theory and established models of health behavior adoption. For instance, the ability to choose elements within the VR intervention aligns with principles of self-determination theory by potentially enhancing autonomy and competence through interactive engagement with the Possible Worlds bubbles [49]. Similarly, the intervention incorporates aspects of the transtheoretical model of change [50] by targeting the precontemplation and contemplation stages through visualization of recovery-related benefits. Given the acute nature of the ED setting, such questions will likely be best addressed through collaboration with laboratory-based human testing. Pragmatic considerations will ultimately include conducting a cost-benefit analysis comparing the VR-Choice platform with more established (but, as noted, potentially insufficient) approaches, such as motivational interviewing, contingency management, and peer recovery coaching, to highlight the unique contribution of the VR approach. Future studies may also wish to examine combining VR-Choice with more traditional approaches for a potential synergistic impact.

The potential applications of this intervention are wide, including acute treatment programs (detoxification centers), outpatient intake assessments, or harm reduction sites, with appropriate modifications to address the specific barriers relevant to each context. In addition to applications for substance use, it may be valuable to explore the use of such motivationally oriented VR experiences in other health behavior change contexts, such as smoking cessation, eating disorders, and physical activity promotion.

### Conclusions

In this paper, we present VR-Choice, a virtual environment designed to improve affect and motivation among patients with OUD in the ED following an overdose and to increase their readiness to engage in shared decision-making for treatment with MOUDs. While the efficacy of this app has not yet been tested, initial feedback from participants with LE points to the feasibility of VR as a way to engage patients undergoing an extremely difficult experience in a challenging environment. This work contributes to the growing body of innovative nonpharmacological approaches for addressing the opioid crisis. Our VR intervention should be positioned within the spectrum of novel digital health interventions being developed for substance use disorders, highlighting how it addresses specific gaps in existing approaches for engaging patients in the critical ED setting.

## Supplementary material

10.2196/84397 Multimedia Appendix 1 Script heard by participants during the virtual reality simulation.

## Abbreviations

ED: emergency department
EIC: emotion-imbued choice
LE: lived experience
MOUD: medications for opioid use disorder
OUD: opioid use disorder
SDM: shared decision-making
VR: virtual reality
