# Supplementary material for: A Virtual Reality Intervention to Promote Uptake of Medications for Opioid Use Disorder in the Emergency Department Following Opioid-Involved Overdose Based on Input From People With Lived Experience: Development Study
Source: JMIR XR Spat Comput. 2026 Jul 6;3:e84397. doi: 10.2196/84397 (PMC13335950; doi:10.2196/84397)
Supplement: Multimedia Appendix 1 [file xr-v3-e84397-s001.docx]

## Appendix A

This appendix contains the script participants heard while in the virtual reality simulation.

### Script:

### Beginning Voiceover

“As you begin your journey, take a moment to sit back, take a few deep breaths, and listen to the sounds around you.”

### Bubble Room Voiceover

“Touch a bubble to choose an immersive scene that looks enjoyable to you. Please experience at least three bubbles, but feel free to explore as many as you’d like. You can also revisit any bubble you’ve already explored. When you are ready to finish, touch the exit bubble to end the program.”

### Exit Bubble Voiceover

“Thank you for participating. Please remove the headset.”
